# The tandem repeat modules of *Xist* lncRNA: a swiss army knife for the control of X-chromosome inactivation

**DOI:** 10.1042/BST20210253

**Published:** 2021-12-09

**Authors:** Ana Cláudia Raposo, Miguel Casanova, Anne-Valerie Gendrel, Simão Teixeira da Rocha

**Affiliations:** 1 Departamento de Bioengenharia e Instituto de Bioengenharia e Biociências, Instituto Superior Técnico, Universidade de Lisboa, Lisbon, Portugal; 2 Associate Laboratory i4HB - Institute for Health and Bioeconomy, Instituto Superior Técnico, Universidade de Lisboa, Lisbon, Portugal; 3 Instituto de Medicina Molecular João Lobo Antunes, Faculdade de Medicina, Universidade de Lisboa, Lisbon, Portugal

**Keywords:** chromatin, epigenetics, lncRNA, tandem repeat, X-chromosome inactivation

## Abstract

*X-inactive-specific transcript* (*Xist*) is a long non-coding RNA (lncRNA) essential for X-chromosome inactivation (XCI) in female placental mammals. Thirty years after its discovery, it is still puzzling how this lncRNA triggers major structural and transcriptional changes leading to the stable silencing of an entire chromosome. Recently, a series of studies in mouse cells have uncovered domains of functional specialization within *Xist* mapping to conserved tandem repeat regions, known as Repeats A-to-F. These functional domains interact with various RNA binding proteins (RBPs) and fold into distinct RNA structures to execute specific tasks in a synergistic and coordinated manner during the inactivation process. This modular organization of *Xist* is mostly conserved in humans, but recent data point towards differences regarding functional specialization of the tandem repeats between the two species. In this review, we summarize the recent progress on understanding the role of *Xist* repetitive blocks and their involvement in the molecular mechanisms underlying XCI. We also discuss these findings in the light of the similarities and differences between mouse and human *Xist*.

## Introduction

*X-inactive-specific transcript* (*Xist*) is a long non-coding RNA (lncRNA) responsible for X-Chromosome Inactivation (XCI), the dosage compensation mechanism that equalizes X gene dosage between XX females and XY males in placental mammals. The importance of *Xist* in XCI regulation was uncovered by loss of function studies showing that lack of dosage compensation for the X chromosome results in female-specific lethality in mice [1,2]. These pioneer studies unraveled a relevant function for this non-coding transcript with implications on survival, placing *Xist* at the central stage of lncRNA research.

*Xist* is the only gene expressed exclusively from the inactive X chromosome (Xi). This monoallelic expression is established through the concerted action of several *cis*- and *trans*-acting players during early embryonic development (reviewed in [3]). *Xist* characteristically covers the Xi in a process that is essential for chromosome-wide transcriptional silencing, formation of facultative heterochromatin and spatial 3D structural rearrangement of the X chromosome (reviewed in [4,5]). How a single lncRNA coordinates these major structural and transcriptional changes over an entire chromosome still remains enigmatic. XCI continues to be a stimulating research field and a ground for prolific experimentation that is constantly re-shaping our understanding of *Xist* function.

**Figure 1.**
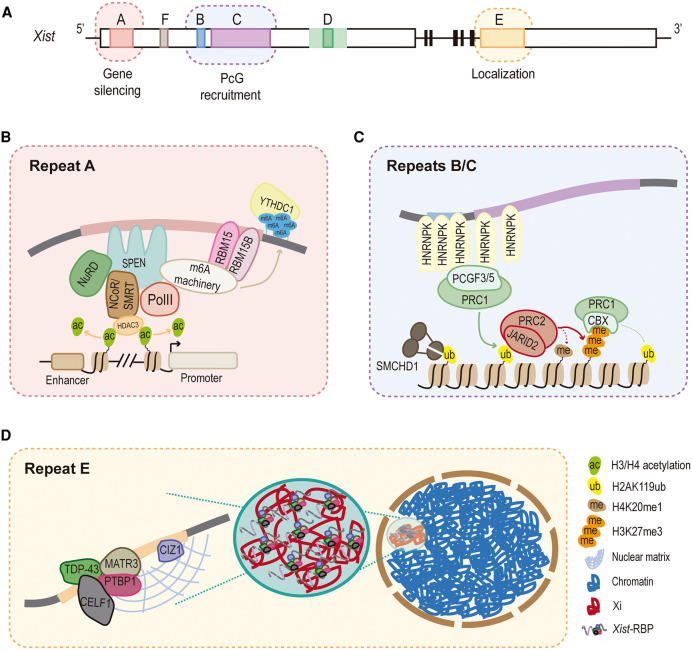
The role of mouse *Xist* repeats in XCI. (**A**) Schematic representation of the mouse *Xist* gene with exons 1-to-7 represented by white and black rectangles, and the Repeats A-to-F highlighted in colors. For Repeat D, both the core motif (dark green) and truncated motifs (light green) are represented. (**B**–**D**) Detailed outline of the factors interacting with Repeat A and mediating gene silencing (**B**), with Repeats B/C and involved in PcG recruitment (**C**), and with Repeat E and important factors for *Xist* localization within the inactive X territory (**D**); refer to main text for details.

The *Xist* gene produces a spliced ∼17-to-19 kb lncRNA. A remarkable feature of *Xist* is the existence of several conserved tandem repeat sequences, known as Repeats A-to-F (Figure 1A). Despite their conservation, considerable variability in the number of repetitive motifs can be found in different mammalian species [6,7]. Whether this variation translates into the functional diversity of *Xist* function across mammals remains an open question. Interestingly, deletion analyses of mouse *Xist* repeats uncovered particular functions of these modules, creating a new paradigm of *Xist* as a multitasking lncRNA with specialized functional domains that interact with specific RNA Binding Proteins (RBPs) [8–11]. Together, these functional studies reveal that the different *Xist* repeats seem to act in a synergistic and coordinated manner, orchestrating the process of XCI from its inception to its end. In this review, we summarize recent progress in our understanding of the role of the different *Xist* repeats and their protein interactors in XCI in the mouse system and discuss how these findings compare to what we know about the human *XIST* RNA.

## Repeat A — The hub for transcriptional silencing

Repeat A, mapping to the first ∼335–700 nucleotides of mouse *Xist* (Figure 1A), is the most conserved RNA module [6,7], composed of seven and a half repetitive motifs that form hairpin-like structures separated by single-stranded regions [12]. Work from several groups, using deletion analysis in murine embryonic stem cells (ESCs) and mouse models, demonstrated that Repeat A is essential for the transcriptional silencing of virtually all genes on the Xi [10,13–17] (Table 1).

**Table 1:** List of *Xist* deletions targeting *Xist* Repeats A-to-F in mouse cells and their associated phenotypes

Repeats	*Xist* RNA deletion	System	Phenotype	References
Repeat A	ΔSX: 5′ end of *Xist* including Repeat A (∼900 bp)	Mouse XY ESCs with an inducible *Xist* at the endogenous locus	Mild effect on *Xist* coating; Gene silencing failure; Recruitment of PcG complexes	[8,17,31]
	ΔA (∼800 bp)	Mice with a Repeat A deletion at the endogenous locus	Loss of *Xist* expression; Gene silencing failure in embryonic and extraembryonic lineages	[14]
	5′ end of *Xist* containing Repeat A (∼900 bp)	Mice with a CAG promoter driving *Xist* expression at the endogenous locus	Gene silencing failure for most, but not all, genes in embryonic and extraembryonic lineages	[15]
	ΔA (∼370 bp)	Mouse XY ES cells with inducible *Xist* cDNA TG in an autosome	Down-regulation of *Xist* expression; Gene silencing failure	[16]
	5′ end of *Xist* including Repeat A (∼725 bp)	Mouse XX Cast/129 hybrid ESCs with an inducible *Xist* at the endogenous locus	Gene silencing failure for most genes	[10]
	ΔA (∼550 bp)	Mouse XX hybrid Cast/129 ESCs with a *Tsix* deletion	Mild effect on *Xist* coating; Gene silencing failure	[13]
Repeats B and C	ΔXN: deletion of Repeats F, B and C (∼4000 bp)	Mouse XY ESCs with an inducible *Xist* cDNA TG integrated in X-linked *Hprt* locus	Complete loss of H3K27me3 and H2AK119ub deposition; Mild reduction in gene silencing; Smaller *Xist* clouds	[17,31]
	ΔXN: deletion of Repeats F, B and C (∼4000 bp)	Mouse ESCs with inducible *Xist* cDNA TG on an autosome	No H3K27me3/H2AK119ub enrichment	[32]
	ΔXR-PID: deletion of Repeat B and a small part of Repeat C (∼600 bp)	Mouse XY Cast/129 hybrid ESCs with an inducible *Xist* TGs on autosomes	No H3K27me3/H2AK119ub enrichment; Reduction in gene silencing	[19]
	ΔF + B + C (∼3900 bp)	Mouse XY ESCs with an inducible *Xist* at the endogenous locus	No H3K27me3/H2AK119ub enrichment	[8]
	ΔB + 1/2C (∼1300 bp)		Significant decrease in H3K27me3/H2AK119ub enrichment	
	ΔB (∼330 bp)		Decrease in H3K27me3/H2AK119ub enrichment	
	ΔC (∼1700 bp)		Normal H3K27me3/H2AK119ub enrichment	
	ΔB + C (∼2100 bp)		No H3K27me3/H2AK119ub enrichment; Relaxation of X-linked gene silencing	
	ΔPID: Repeat B and partial repeat C (∼1700 bp)	Mouse XX Cast/129 hybrid ESCs with an inducible *Xist* at the endogenous locus	No H3K27me3/H2AK119ub enrichment; Reduction in gene silencing	[10]
	ΔB (∼300 bp)	Transformed tetraploid female MEFs	Diffuse *Xist* clouds; No H3K27me3/H2AK119ub enrichment	[9]
	ΔC (∼1700 bp)		Normal H3K27me3/H2AK119ub enrichment	
	ΔB (∼300 bp)	Mouse XX hybrid Cast/129 ESCs with a *Tsix* deletion	Initial H3K27me3 and H2AK119ub enrichment is not maintained; Reduction in gene silencing; Diffuse *Xist* clouds	[9,13]
	ΔB (∼300 bp)	Mouse XX hybrid Cast/129 ESCs with a MS2-tagged 129 *Xist* allele	Loss of Xi compaction; Impaired inactivation of late silenced genes	[41]
Repeat E	ΔE (∼1300 bp)	Mouse XX hybrid Cast/129 ESCs with a *Tsix* deletion	Increase expression of escape genes	[48]
	ΔE (∼1500 bp)	Mouse XY ESCs with inducible *Xist* cDNA TG in an autosome	No CIZ1 recruitment	[46]
	ΔE (∼1200 bp)	Transformed tetraploid female MEFs	Disruption of *Xist* coating; Reduced H3K27me3 enrichment; No CIZ1 recruitment	[47]
	ΔE (∼1300 bp)	Mouse XX hybrid Cast/129 ESCs with MS2-tag on *Xist* 129 allele	Normal initiation of XCI; *Xist* RNA dispersal from the Xi, defective gene silencing and decreased H3K27me3 enrichment at the maintenance stage	[11]
Repeat F	X-FR mutant: deletion including Repeat F (∼700 bp)	Transformed tetraploid female MEFs with an inducible *Xist* transgene on an autosome	Reduced *Xist* expression and weak *Xist* clouds	[53]
	ΔLBS region: deletion including Repeat F (∼785 bp)	Mouse XY ESCs with inducible *Xist* expression from the endogenous locus	Compromise recruitment of the Xi to the nuclear lamina; Gene silencing failure	[52]
	Deletion containing Repeat F (∼950 bp)	Transformed tetraploid female MEFs	Reduced *Xist* expression and weak *Xist* clouds	[9]
	ΔLBS region: deletion including Repeat F (∼850 bp)	Mouse XX Cast/129 hybrid ESCs with an inducible *Xist* at the endogenous locus	Weak defect in gene silencing	[10]
Repeat D	ΔD (∼1100 bp)	Transformed tetraploid female MEFs	No effect o*n Xist* coating or PcG recruitment	[9]

Abbreviations: CAG, Cytomegalovirus-Actin-Globin; Cast/129, Mus musculus Castaneus/Mus musculus domesticus 129 strain; cDNA, complementary DNA; CIZ1, CDKN1A-Interacting Zinc finger protein 1; ESCs, Embryonic Stem Cells; H2AK119ub, ubiquitination of the histone H2A at lysine 119; H3K27me3, tri-methylation of histone H3 on lysine 27; *Hprt*, Hypoxanthine phosphoribosyltransferase; MS2, Bacteriophage MS2 (Emesvirus zinderi); TG, TransGene; *Tsix*, *Xist* antisense RNA; Xi, inactive X chromosome; *Xist*, X-*i*nactive-*s*pecific transcript; XX: female biological sex; XY, male biological sex.

The mechanistic understanding of the transcriptional silencing imposed by Repeat A has evolved considerably in the past decade. An initial model postulated that transcriptional silencing was achieved through the interaction with Polycomb Repressive Complex 2 (PRC2), a repressive modifying chromatin complex responsible for the tri-methylation of histone H3 on lysine 27 (H3K27me3) that is enriched along the Xi [18]. This model was challenged after the identification of a different RNA module as the main responsible for PRC2 recruitment (Repeats B/C, see below) [8–10,19]. In addition, the discovery of bona-fide Repeat A interactors by unbiased proteomic approaches and mapping of RNA–protein interactions [12,20,21] allowed a more precise understanding of the molecular function of this module in XCI, which does not involve PRC2.

Among the Repeat A interactors, SPEN (SPlit ENds), a >400 kDa RBP containing a SPOC (SPEN Paralogue/Orthologue C-terminal) transcriptional repressive domain, has been identified as a major player in *Xist*-dependent transcriptional silencing [20,22–25]. Interaction of SPEN with Repeat A is dependent on its RNA Recognition Motifs (RRM) 2–4 [12,20,22,25] that are important to ensure *Xist* stability and spreading across the X chromosome undergoing inactivation [26]. Upon interaction with *Xist,* SPEN is recruited to both active promoters and enhancers on the X chromosome to effectively initiate gene silencing [22]. At these sites, SPEN, through its SPOC domain, acts as a scaffold to recruit repressive factors such as the NCoR/SMRT (Nuclear receptor Co-Repressor/ Silencing Mediator of Retinoic acid and Thyroid hormone receptor) complex [22]. Through its HDAC3 (Histone DeACetylase 3) subunit, the NCoR/SMRT complex is responsible for deacetylating histones H3 and H4, one of the first chromatin events during XCI [27]. However, lack of HDAC3 only has a mild impact on gene silencing [22,27], suggesting that other SPEN partners are involved in the transcriptional silencing of the Xi. Indeed, the SPOC domain of SPEN also interacts with the NuRD (Nucleosome Remodeling and Deacetylase) complex, RNA polymerase II (PolII) and associated factors, as well as the N6-methyladenosine (m6A) RNA methylation machinery [22], but the relevance of these interactions for XCI are yet to be understood (Figure 1B).

Additional SPOC-containing proteins, such as RBM15 (RNA Binding Motif protein 15) and RBM15B also interact with Repeat A [20,21,23]. These proteins play a central role in the recruitment of the m6A RNA methylation machinery. m6A is the most abundant modification in eukaryotic RNAs, influencing several RNA processing events such as stability, splicing, translation and secondary structures, in both physiological and pathological contexts (reviewed in [28]). m6A is deposited along the *Xist* transcript, which includes a m6A hotspot just downstream of Repeat A [21,29]. This modification is then recognized and bound by the YTHDC1 (YTH Domain Containing 1) m6A reader [21,29,30] (Figure 1B). The involvement of RBM15/15B, the m6A methylation machinery and YTHDC1 were initially suggested to have an important impact on X-linked gene silencing [21]. However, according to recent data, the role of RBM15/15B, the m6A methylation complex or the m6A hotspot region downstream of Repeat A seem to be rather modest [10,30] and clearly do not compensate for the effects of SPEN loss [22]. Further experiments will be needed to have a finer understanding of the role of m6A methylation in XCI. To summarize, Repeat A acts as a hub for the initiation of transcriptional silencing by recruiting SPEN, other SPOC-containing proteins and associated repressive factors to elicit gene silencing as *Xist* spreads along the X chromosome (Figure 1B).

## Repeats B/C — The hub for recruitment of Polycomb group complexes

The region of mouse *Xist* containing Repeats B and C folds independently from the Repeat A module [29]. Repeats B and C have a very different sequence content, but both are cytosine-rich. Whereas Repeat B is composed of ∼30 CCCCAG/CCCCUG motifs mapping to nucleotides ∼2850–3050, Repeat C is composed of 14 sequence motifs of ∼115 bp mapping to nucleotides ∼3115–4730 in the mouse *Xist* transcript. The Repeats B/C module is important for locking the silent state of the Xi, through the recruitment of Polycomb Group (PcG) protein complexes to the Xi upon initial establishment of *Xist*-mediated silencing by the Repeat A/SPEN module.

The first hint for an involvement of these repeats in PcG protein recruitment came from the observation that an inducible *Xist* cDNA transgene lacking Repeats B and C, along with Repeat F, called ΔXN, is unable to recruit the PRC2 complex to the Xi, in contrast with a *Xist* mutant lacking the Repeat A (ΔA) [31]. This region was then shown to be also required for the recruitment of the Polycomb Repressive Complex 1 (PRC1), responsible for ubiquitination of the histone H2A at lysine 119 (H2AK119ub) [32]. Further deletion analysis mapped the minimal sequence for PRC1/PRC2 recruitment to a region containing Repeat B and a portion of Repeat C [19]. When individually deleted, Repeat B has a notorious impact on PcG recruitment, in contrast with the absence of a measurable effect of Repeat C [8,9]. However, PcG protein recruitment to the Xi is only found to be completely abolished when both Repeats B and C (or a substantial part of Repeat C) were missing [8,10,13] (Table 1).

How the B/C module recruits both PRC1 and PRC2 to the Xi and the corresponding sequence of events are questions that have been widely investigated. Work from different labs showed that cytosine-rich Repeats B and C act as docking sites for HNRNPK (Heterogeneous Nuclear RiboNucleoProtein K) [8,9,19,33,34]. HNRNPK interacts with the non-canonical PCGF3/PCGF5–PRC1 complex that is brought to the Xi, decorating chromatin with the H2AK119ub mark [9,19]. This chromatin modification precedes H3K27me3 enrichment on the Xi [27] and serves as the main mechanism to recruit PRC2 through the recognition of H2AK119ub by the JARID2 cofactor [31,35,36]. The H3K27me3 mark will then serve as an anchor for the recruitment of the CBX (ChromoBoX homolog) subunit of canonical PRC1 [37], thus reinforcing the PcG-mediated heterochromatinization of the Xi (Figure 1C). This is recognized as the major pathway for PcG protein recruitment to the Xi by the B/C module, but alternative mechanisms have also been proposed [9].

The dynamics of PcG recruitment mediated by Repeats B/C follows *Xist* coating over the Xi and is initially observed at intergenic regions [8,10,27]. Subsequent spreading to active genes is only observed following transcriptional silencing [27]. The dynamics of accumulation of PcG marks over active genes could be attributed to a direct role of Repeat A in PcG recruitment to genes [13] or simply be a secondary event to the transcriptional silencing imposed by the Repeat A/SPEN pathway. We favor this second hypothesis given that the transcriptional silencing of CG-rich promoters, such as the ones for many X-linked genes, creates a chromatin environment permissive for PRC2 recruitment [38–40]. In this respect, mapping H3K27me3/H2AK119ub enrichment over the Xi in *Spen* SPOC mutants will be important to disentangle between a direct or a passive role for Repeat A in PcG recruitment to X-linked genes undergoing silencing.

Whereas Repeat A seems to be the main actor on the initial steps of XCI, Repeats B and C appear to take the center stage once XCI has been set in motion. Accordingly, X-linked gene silencing is initiated but is partially defective in *Xist* ΔB/C mutants [8–10,41] (Table 1). Likewise, removal of PCGF3/PCGF5–PRC1, recruited by Repeats B/C, also causes reduced *Xist*-mediated gene repression [10,32]. This important role of Repeats B/C in the maintenance of XCI, may depend on the involvement of PcG proteins on *Xist* spreading over the Xi [9], but also on several redundant mechanisms of repression enabled by PcG protein recruitment. For instance, Repeats B/C are important for the deposition of the H4K20me1 heterochromatin mark, presumably secondary to PRC2 recruitment [37,42] (Figure 1C). The B/C module also allows the recruitment of the structural protein SMCHD1 (Structural Maintenance of Chromosomes flexible Hinge Domain containing 1), downstream of the PCGF3/PCGF5–PRC1 complex [43] (Figure 1C). SMCHD1 is fundamental for promoting the characteristic folding of the Xi in two mega domains largely depleted of Topologically Associating Domains (TADs) [44,45]. A recent study also implicated Repeat B in this module in Xi compaction [41]. Whether other hallmarks of the Xi, such as recruitment of the macroH2A histone variant, DNA methylation of gene promoters are dependent on Repeats B/C remain to be tested. In summary, by recruiting PcG complexes, Repeats B/C play a key role in heterochromatin formation and topological reconfiguration of the X chromosome, being essential for long-lasting transcriptional silencing along the entire Xi.

## Repeat E — The hub for *Xist* localization to the inactive X territory

Repeat E is localized at the beginning of exon 7 and maps to nucleotides ∼10 275–11 400 of mouse *Xist* transcript. It is composed of two types of tandem repeats: around 35 C/U/G-rich motifs of ∼16–27 bp at the 5′end and ∼25 C/U-rich motifs of 6–19 bp towards its 3′end. Different *Xist* ΔE deletions invariably result in delocalization of *Xist* from the Xi territory that aggravates as cells enter the maintenance stage of XCI [11,46–48] (Table 1). Repeat E is not necessary for initiating X-linked gene silencing or PcG recruitment, but the inability of *Xist* ΔE to coat the X chromosome ultimately results in gene reactivation and loss of PcG marks at the maintenance stage of XCI [11].

Several proteins were shown to bind Repeat E and to be important for tethering *Xist* to the Xi territory. One of the first interactors identified was CIZ1 (CDKN1A-Interacting Zinc finger protein 1). CIZ1 is a component of the nuclear matrix, a filamentous insoluble protein network that appears to participate in restricting *Xist* RNA molecules to the Xi [46,47] (Figure 1D). More recently, several RBPs, such as PTBP1, MATR3, CELF1, and TDP-43 were shown to directly interact with Repeat E [11] (Figure 1D). They form a distinct functional complex that assembles at Repeat E independently of CIZ1, being important to stabilize *Xist* coating after the initial wave of transcriptional silencing and PcG recruitment by Repeat A and Repeats B/C, respectively [11]. These proteins, namely PTBP1 and MATR3 have the capacity to form liquid droplets *in vitro* [49,50]. In particular, PTBP1 was shown to form phase-separated droplets upon binding to the Repeat E module [11]. This observation suggests that Repeat E may be important for the assembly of a specialized phase-separated sub-nuclear compartment necessary for the efficient maintenance of XCI [11,51]. This is an attractive idea to explain the remarkably stable epigenetic silencing of the Xi once XCI is established. Whether a Xi phase-separated condensate is formed and whether this biophysical compartment plays a role in the maintenance of XCI still awaits experimental confirmation. Overall, Repeat E interacts with several RBPs, which are essential for anchoring *Xist* RNA molecules to the Xi territory (Figure 1D), playing a critical role in the maintenance of XCI.

## Other relevant repeat domains in *Xist*

The other two repetitive modules, Repeats F and D have attracted less attention. Repeat F is the shortest of all repeats and is made of two copies of a 10 bp motif (UGGCGGGCUU) mapping to nucleotides ∼1540–1575 in the mouse *Xist* RNA. Studies addressing the role of Repeat F resorted so far to larger deletions that extended beyond this module [9,10,52,53] (Table 1). The deletion of this region, also known as LBS, disrupts several binding sites for YY1 (Yin and Yang 1), a transcription factor important for *Xist* expression [54,55] and interferes with the binding of LBR (Lamin B receptor) in the vicinity of Repeat F, which consequences for XCI remain conflicting [10,52]. It is important to consider that such deletions can interfere with important DNA regulatory elements for XCI, besides having a direct influence on *Xist* RNA, a phenomenon that has previously been documented for some ΔA mutants [14,16] (Table 1). To disentangle between these, a study using inducible *XIST* from its endogenous locus in male ESCs shows that deletion of this region has a major impact on transcriptional silencing due to loss of tethering of the Xi to Lamin B at the nuclear periphery [52]. In contrast, a recent report using a similar inducible system in female ESCs shows that deletion of the LBS region only has marginal effects on gene silencing [10]. A precise deletion of Repeat F will be necessary to unveil its function in XCI.

The Repeat D is the least characterized repetitive module of mouse *Xist*. Originally, a single ∼200 bp core motif was identified in mouse *Xist* [56], but up to 10 truncated Repeat D motifs, located at nucleotides ∼5200–7900 towards the 3′ end of exon 1, was later described [6] (Figure 1A). The only ΔD deletion described, that includes the core motif, shows no noticeable phenotypes in terms of *Xist* coating and PcG recruitment in Mouse Embryonic Fibroblats (MEFs) [9] (Table 1). This does not exclude a role for Repeat D in the initial stages of XCI or when the full 3 kb region is deleted. It is however important to reiterate that Repeat D in rodents is heavily degenerated in contrast with other mammals, suggesting that deletion analysis of the mouse Repeat D might not be relevant for other mammalian species.

## Mouse versus human *Xist*

Most of our understanding about the role of *Xist* in XCI comes from the mouse model. However, an increasing body of evidence has defied the murine-centric view of XCI, revealing that many of the developmental and molecular features observed in mice are not conserved in humans. Three major differences stand out between mouse and human XCI (reviewed in [57,58]): in the mouse preimplantation embryo, an initial form of paternally imprinted XCI takes place, which is maintained in extraembryonic lineages but reversed in the embryo proper; in human blastocysts, *XIST* is expressed from both active X chromosomes, prior the onset of random XCI; the number of genes escaping silencing in humans is remarkably higher than in mice. Whether these differences reflect a distinct species-specific modular organization of the *Xist* molecule is only starting to be dissected.

**Figure 2.**
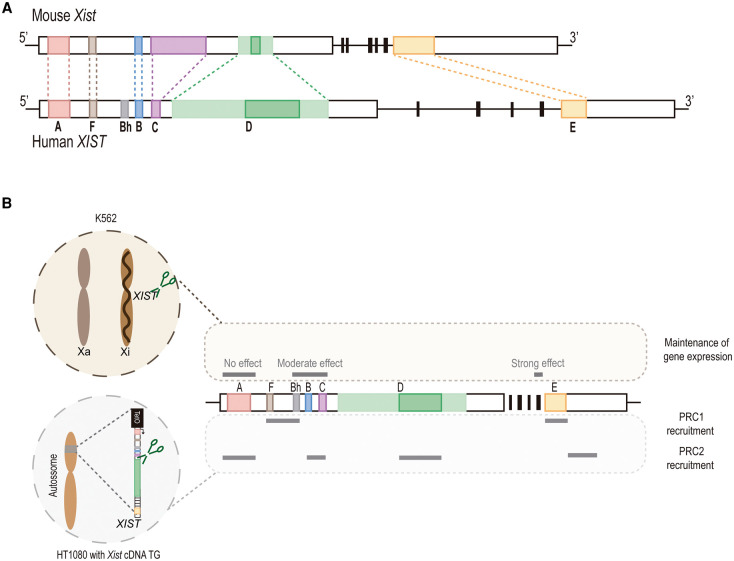
Similarities and differences between mouse and human *Xist*. (**A**) Comparison between mouse and human *Xist* gene structure and tandem repeats. Core Repeat D and its extended truncated motifs are marked in dark and light green, respectively. (**B**) Outline of the putative role of human *XIST* repeats for XCI maintenance assessed in female K562 lymphoblast cell line and for XCI initiation in male HT1080 fibrosarcoma cell line with a Xist cDNA autosomal transgene; see main text for details.

Overall, the gene structure of *Xist* is conserved between mouse and humans (Figure 2A). Repeats are amongst the most conserved blocks, in particular Repeats A, E and F, but some important differences in the number of repetitive motifs are seen for the other repeats. For instance, Repeat B is duplicated in human *XIST*, with an extra B-like repeat, known as Bh, located between Repeat F and B. Repeats C and D are the ones that differ the most between mouse and human *Xist*. While Repeat C is considerably expanded in the mouse, a feature that seems to be rodent-specific, it is reduced to a single motif in humans. In contrast, Repeat D is considerably expanded in human *XIST,* while it is mostly made of truncated motifs in rodent lineages [6,7] (Figure 2A).

Despite these sequence differences, the higher-order folding of *Xist* RNA was shown to be conserved between mouse and human, being partitioned in five major structural modules: Repeats A, F, B/C/D, E and exon 6/7 [12,29]. Moreover, mouse and human *Xist* also share an extensive fraction of their protein interactome [20,59]. Proteomic screening of *XIST* interactors in human B lymphocytes by ChIRP-MS (Comprehensive Identification of RNA-binding Proteins by Mass Spectrometry) identified factors known to interact with mouse *Xist*, such as SPEN, RBM15/RBM15B, HNRNPK or CIZ1 as well as novel functional interactors, like TRIM28 (TRIpartite Motif-containing 28, commonly known as KAP1) [59]. Whether TRIM28 is a species- or cell type-specific *XIST* interactor is not yet known. Besides a shared set of protein interactors, these interactors distribute in a similar fashion along the mouse and human *Xist* molecules as revealed by enhanced CLIP-seq (UV Cross-Linking and ImmunoPrecipitation coupled with high-throughput sequencing) [60]. Small differences, namely on the interaction of HNRNPK with the expanded human Repeat D, have been described [29]. This raises the intriguing possibility that the larger Repeat D might replace the function of Repeat C in humans, which is reduced to one incomplete motif. It also suggests that the distribution of RBPs between different repetitive modules might confer slightly different functions to *Xist* in different species.

Recent studies, resorting to segmental deletions of human *XIST* in cancer cell lines, have started to explore the role of the repeats in the initiation and maintenance stages of human XCI, allowing to further uncover similarities and differences with mouse *Xist* [61,62]. In K562 erythromyeloblastoid leukemia cell line, where XCI has already occurred, a homozygous deletion of the entire *XIST* gene results in a global up-regulation of X-linked genes [62]. Several deletions along human *XIST* were also made in K562, many of which result in multiple defects at the transcriptional and splicing levels and/or affect *XIST* coating [62]. For instance, ΔD mutants show decreased RNA levels and splicing abnormalities that result in moderate X-linked reactivation, as previously observed in 293FT cell line [63]. Concentrating on *XIST* mutants with no such defects in K562, it was revealed that exon 5, which does not contain any repeat module, has an important role in XCI maintenance that has not yet been mechanistically explored. In contrast, deletions of Repeat A and Repeat B/C show no or moderate effects on XCI maintenance, respectively, in K562 cells [62] (Figure 2B).

A crucial role for human *XIST* in XCI maintenance in K562 contrasts with the situation in the mouse, where no visible effect on gene silencing is observed when *Xist* or its repeats are deleted in MEFs [9,64]. However, these results must be taken with caution, as this cancer cell line presents several (epi)genetic abnormalities that hinder the robustness of this cellular model to study XCI maintenance. For example, the Xi shows an atypical XCI status, being devoid of PcG marks [62]. As such, it might be over-simplistic to assume that human *XIST*, but not mouse *Xist*, plays a role in the maintenance of XCI. As a matter of fact, the emerging picture is that the role of *Xist* in XCI maintenance is complex and cell-type specific, whether in mouse or human [59,65–68].

A more recent study investigated the initiation phase of human XCI, using inducible autosomal cDNA transgenes in the HT1080 male fibrosarcoma cell line [61]. Hallmarks of facultative heterochromatin of the Xi are recapitulated in this system and were used to identify the regions of human *XIST* essential for H3K27me3 and H2AK119ub accumulation on the Xi. In contrast to mouse ESCs, PRC1 and PRC2 recruitment in HT1080 cells, rely on different *XIST* modules (Figure 2B): Repeats F/Bh and E are important for the accumulation of the PRC2 mark, H3K27me3, while enrichment of H2AK119ub by PRC1 is dependent on Repeats A, B/C, D and the 3′ non-repetitive part of *XIST*. The independent recruitment of PRC1 and PRC2 was further confirmed using chemical inhibition of the catalytic domains of the two PcG complexes. Overall, these results differ remarkably from mouse cells, where PRC2 recruitment is mostly downstream of the *Xist-*mediated recruitment of PCGF3/5-PRC1 through HNRNPK [9,10,32]. However, direct comparison between studies on mouse ESCs and human cancer cells should be taken cautiously, especially when *XIST* anchoring to the Xi differs in primary versus transformed cells [69]. A more appropriate comparison would be to look into *XIST* mutants in human ESCs. Unfortunately, human ESCs are mostly found in a post-XCI or in a culture-induced eroded XCI state (reviewed in [70]). Continuing progress in naïve culture conditions, which should reset human ESCs to a pre-XCI state [71], will be fundamental to reveal similarities and differences between human and mouse *Xist* and their role in the initiation of XCI.

## Concluding remarks

Since its discovery, in the early nineties, *Xist* has been under the spotlight of lncRNA research. Although a myriad of intergenic, intronic and antisense lncRNAs have now been identified, the biological relevance of these molecules has remained challenging to define. In no small measure, our understanding of *Xist* has been central in promoting and inspiring the study of other nuclear-located lncRNAs which, despite being poorly conserved compared with protein-coding genes, share several functions and mechanisms of action. In this sense, the idea that lncRNAs can organize in structural modules, similarly to *Xist*, is a tantalizing hypothesis to explain how lncRNAs develop their tissue- and species-specific roles. Indeed, it was recently shown that *Rsx* (RNA-on-the-silent X), the lncRNA responsible for XCI in marsupials but with no homology to *Xist*, presents multiple tandem repetitive modules reminiscent of *Xist* repeats [72]. Just like *Xist*, *Rsx* might resort to different repetitive modules as functional entities to regulate XCI in marsupials. This example illustrates how further research on *Xist* RNA might have important implications for our understanding of other lncRNAs.

Although the knowledge that has been acquired about *Xist* in the past decades is undeniable, many open questions still remain. For example, the crosstalk between the different RNA modules and their protein interactors still needs to be mechanistically explored. Furthermore, while most studies have focused on dissecting *Xist* function during the establishment phase of XCI, a maintenance role for *Xist* has just started to be unveiled with cell-type-specific characteristics [59,68]. This is particularly relevant in the context of diseases predominantly affecting women for which incorrect maintenance of X-linked gene dosage may play a role, such as auto-immune diseases like systemic lupus erythematosus or multiple sclerosis (reviewed in [73]). A possible link between *XIST* and human disease will certainly be one of the areas to explore in the future.

## Perspectives

- *Xist* lncRNA is the master regulator of X-chromosome inactivation. Discovered thirty years ago, the molecular mechanisms through which *Xist* renders an entire chromosome into a stable inactive state remain enigmatic.
- A series of deletions in mouse cells revealed that *Xist* possesses several modules of functional specialization mapping to the conserved Repeats A-to-F. These functional modules execute specific tasks through interaction with various RNA-binding proteins in a coordinated manner during XCI. This modular organization and multitasking nature of *Xist* is conserved in humans, although the functional specialization of some repeats might have evolved novel functions.
- It still remains to be deciphered the molecular mechanisms underlying the interplay between *Xist* repeats for the control of XCI. Also, the conservation of these events in humans is unknown and will need to be investigated in more appropriate cellular models. Finally, the translation of these findings to other lncRNAs might reveal new molecular insights in the function of these molecules in a vast plethora of biological systems.

## Abbreviations

CBX, Chromobox homolog; CHIRP-MS, comprehensive identification of RNA-binding protein by mass spectrometry; CIZ1, CDKN1A-interacting zinc finger protein 1; CLIP-seq, UV cross-linking and immunoprecipitation coupled with high-throughput sequencing; ESCs, embryonic stem cells; HDAC3, Histone deacetylase 3; HNRNPK, Heterogeneous nuclear ribonucleoprotein K; lncRNA, long non-coding RNA; m6A, N6-methyladenosine; MEFs, mouse embryonic fibroblasts; NCoR/SMRT, Nuclear receptor co-repressor/Silencing mediator of retinoic acid and thyroid hormone receptor; NuRD, Nucleosome remodeling and deacelyase; PcG, Polycomb group; PRC1, Polycomb Repressive Complex 1; PRC2, Polycomb Repressive Complex 2; PolII, RNA polymerase II; LBR, lamin B receptor; RBPs, RNA binding proteins; RBM15, RNA binding motif protein 15; Rsx, RNA-on-the-silent X; RRM, RNA recognition motifs; SMCHD1, Structural maintenance of chromosomes flexible hinge domain containing 1; SPEN, Split Ends; SPOC, SPEN paralogue/orthologue C-terminal; TADs, Topologically associating domains; TRIM28, Tripatite motif-containing 28; XCI, X-chromosome Inactivation; Xi, inactive X chromosome; Xist, X-inactive-specific transcript; YTHDC1, YTH domain containing 1; YY1, Yin yang 1
